# More Than Mortar: Glia as Architects of Nervous System Development and Disease

**DOI:** 10.3389/fcell.2020.611269

**Published:** 2020-12-14

**Authors:** Inês Lago-Baldaia, Vilaiwan M. Fernandes, Sarah D. Ackerman

**Affiliations:** ^1^Department of Cell and Developmental Biology, University College London, London, United Kingdom; ^2^Institute of Neuroscience, Howard Hughes Medical Institute, University of Oregon, Eugene, OR, United States

**Keywords:** glia, nervous system development, neural specification, circuit wiring, circuit function, neurodevelopmental disorders, neurodegenerative disorders

## Abstract

Glial cells are an essential component of the nervous system of vertebrates and invertebrates. In the human brain, glia are as numerous as neurons, yet the importance of glia to nearly every aspect of nervous system development has only been expounded over the last several decades. Glia are now known to regulate neural specification, synaptogenesis, synapse function, and even broad circuit function. Given their ubiquity, it is not surprising that the contribution of glia to neuronal disease pathogenesis is a growing area of research. In this review, we will summarize the accumulated evidence of glial participation in several distinct phases of nervous system development and organization—neural specification, circuit wiring, and circuit function. Finally, we will highlight how these early developmental roles of glia contribute to nervous system dysfunction in neurodevelopmental and neurodegenerative disorders.

## Introduction

Long past the days of being relegated to nervous system “glue,” today glia are acknowledged as essential players in nervous system development and function (Allen and Lyons, 2018). Given that glia-like cells are present in the most evolutionarily ancient bilateria, and share common features and functions across divergent species (Verkhratsky et al., 2019), it is unsurprising that invertebrate and vertebrate genetic model organisms have expanded our understanding of the multifaceted roles that glia play in the developing and mature nervous system (Sieger and Peri, 2013; Ackerman and Monk, 2016; Allen and Eroglu, 2017; Kremer et al., 2017; Singhvi and Shaham, 2019; Yildirim et al., 2019).

**FIGURE 1:**
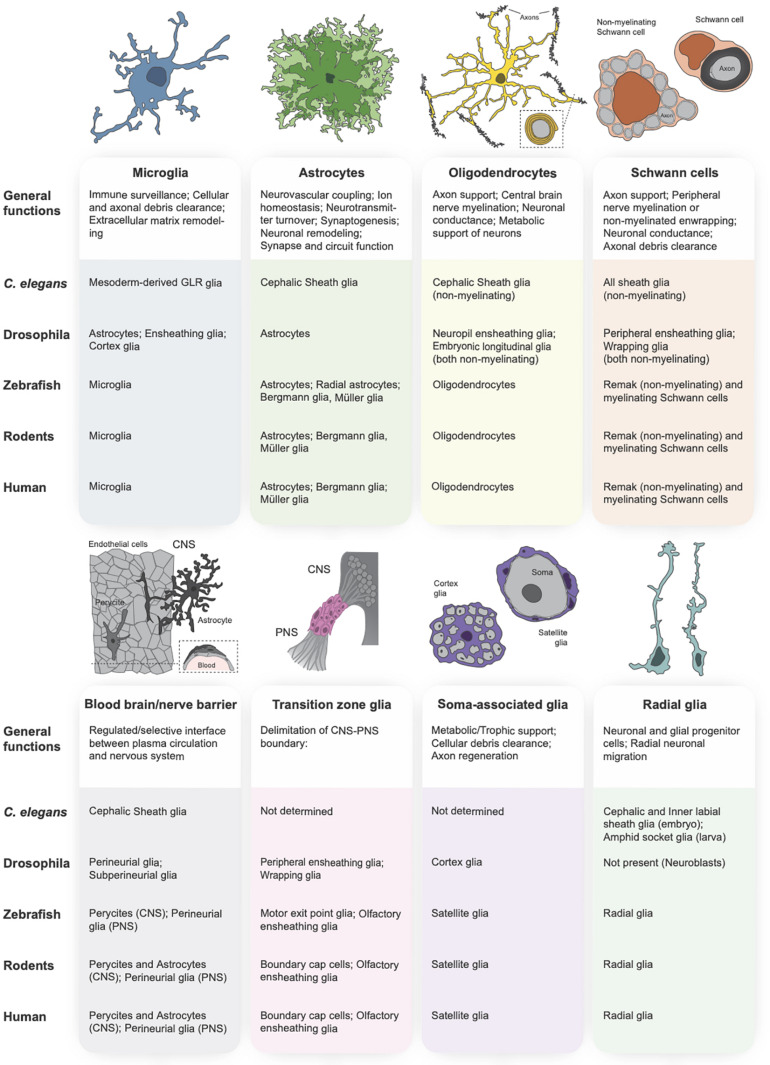
Glial subtypes of the central and peripheral nervous systems. The primary vertebrate glial subtypes can be broken down into the following categories: microglia (schematized in blue), astrocytes (green), oligodendrocytes (yellow), Schwann cells (orange), blood-brain and blood-nerve barrier glia (gray), transition zone glia (pink), soma-associated glial cells (purple), and radial glia (teal). A functional description of each glial subtype is provided below each schematic, followed by a table indicating the orthologous glial cell population in the primary model organisms (*C. elegans*, *Drosophila*, zebrafish, rodent) and in human.

**FIGURE 2:**
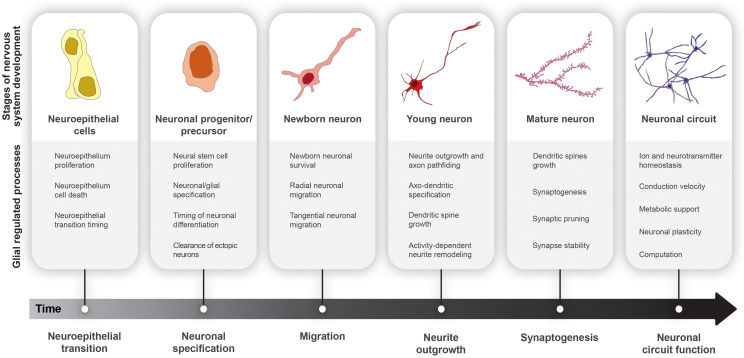
Glial cell functions in nervous system development. Progression from neuroepithelial cells (yellow) to functional neural circuits (purple) over developmental time.

Glial cell populations can be subdivided based on lineage and function, most of which are conserved from invertebrates through humans (Figures 1, 2). Dysfunction of these glial populations has been documented in various neurological disorders, including Huntington’s disease, glioma, and autism (Phatnani and Maniatis, 2015; Hambardzumyan et al., 2016; Dulamea, 2017; Liddelow and Barres, 2017; Neniskyte and Gross, 2017; Hickman et al., 2018; Santosa et al., 2018), but whether glial cell impairments are causative or simply symptomatic of pathology is not yet understood. We propose that characterizing glial function under normal physiological conditions will inform our understanding of the etiology of neurological disorders, which is essential for successful therapeutic intervention. In this review, we will highlight findings from vertebrate and invertebrate systems that focus on roles of glia in nervous system development, and discuss how defects in these processes may contribute to neurodevelopmental and neurodegenerative disease.

## Glia Regulate Brain Size and Neuronal Numbers

Nervous systems are highly heterogeneous structures, containing more unique cell types than any other organ. During development, neuroepithelial cells expand through symmetric mitoses before being converted into neural stem cells (NSCs). These neural stem cells (called neuroblasts in *Drosophila*) must divide for a stereotyped period of time. Their progeny, postmitotic neurons and glia, must then adopt distinct fates in proper numbers and stoichiometry before connecting with the appropriate partners to form functional circuits (McKay, 1989; Brody and Odenwald, 2002; Riccomagno and Kolodkin, 2015; Silbereis et al., 2016; Beattie and Hippenmeyer, 2017; Doe, 2017; Li et al., 2018). Our ability to process sensory information and perform complex tasks depends critically on the timely generation and wiring of diverse neural subtypes into functional circuits. Accordingly, altering neuronal numbers, stoichiometry, diversity, and connectivity all have profound impacts on brain function.

### Glial Regulation of Neuroepithelial Expansion and Transition

In vertebrates, NSCs of the developing brain and spinal cord are called radial glia (Doetsch et al., 1999; Malatesta et al., 2000; Noctor et al., 2001; Kriegstein and Alvarez-Buylla, 2009). During development, radial glia first enter a neurogenic phase to generate neurons, and then switch to a gliogenic phase for production of oligodendrocytes and astrocytes (Malatesta and Götz, 2013; Götz et al., 2016). In contrast, the production of post-mitotic neurons and glia occurs in parallel in *Drosophila* (Homem and Knoblich, 2012). Accordingly, *Drosophila* glial cells form an important component of the NSC niche that regulates neurogenesis (Doe, 2017). This has been well-studied within the *Drosophila* visual system, which arises from a specialized neuroepithelium during development called the outer proliferation center (OPC). This neuroepithelium is located at the surface of the developing optic lobe, where it generates the neurons of the medulla and lamina neuropils. The OPC proliferates and expands during the first and second larval instars before a proneural wave sweeps across the epithelium, resulting in a switch to asymmetric division for generation of neuroblasts (Doe, 2017; Holguera and Desplan, 2018). Immature cortex glia sit atop the OPC and send extrinsic cues to set the balance between neuroepithelial expansion and neuroblast transition. Cortex glia activate Notch signaling in neuroepithelial cells through the membrane-bound ligand Serrate, which maintains epithelial cell proliferation and delays proneural wave progression (Pérez-Gómez et al., 2013). Cortex glia also secrete the Epidermal Growth Factor (EGF) Spitz, which activates EGF Receptor (EGFR) signaling in the epithelium; EGFR activity inhibits Notch to promote proneural wave progression and the transition to neuroblasts (Morante et al., 2013). The relative strength of Notch and EGF signaling within the neuroepithelium governs the timing of this transition (Egger et al., 2010; Yasugi et al., 2010). What controls the balance of these two glial-derived cues is not yet understood. More recently, expression of the chloride channel CIC-a in cortex glia was also shown to promote neuroepithelial expansion, suggesting that glia-mediated ion homeostasis within the niche can temporally control the transition to neurogenesis (Plazaola-Sasieta et al., 2019).

### Glia and the Timing of Neural Progenitor Proliferation

In *Drosophila*, glia can secrete factors to regulate neuroblast proliferation and consequently, neuronal numbers. For example, glial-secretion of the glycoprotein Anachronism represses neuroblast proliferation (Ebens et al., 1993), whereas surface localization of the heparan sulfate proteoglycan Dally-like (Kanai et al., 2018) or E-cadherin (Dumstrei et al., 2003) promotes neuroblast proliferation. The best characterized instance of glia regulating neuroblast proliferation is that of neuroblast reactivation following quiescence. At the end of embryogenesis, neuroblasts enter quiescence and later reactivate in response to feeding during larval life. Neuroblast re-activation requires a nutrient-dependent fat-body-derived signal (Britton and Edgar, 1998; Chell and Brand, 2010; Sousa-Nunes et al., 2011; Yuan et al., 2020). Surface and cortex glia receive this signal, and in response, secrete Insulin-like peptides to reactivate quiescent neuroblasts (Chell and Brand, 2010; Sousa-Nunes et al., 2011; Yuan et al., 2020). A similar mechanism of glial relay through Insulin Receptor signaling was described for the differentiation of lamina neural precursors in the optic lobe, which is necessary for the correct formation of the retinotopic visual system (Fernandes et al., 2017).

Macroglia (astrocytes and oligodendrocytes) are not present during vertebrate neurogenesis in the embryonic central nervous system (CNS). In contrast, microglial populations migrate from the yolk sac into CNS during early embryonic development (Sominsky et al., 2018), and are therefore present at the correct time and place to modulate neurogenesis (Aarum et al., 2003; Antony et al., 2011). Several key *in vitro* studies using primary murine neural progenitor cells were the first to demonstrate the ability of microglia to influence developmental neurogenesis. First, both embryonic and adult neural stem cells could migrate along gradients of microglia-conditioned medium (Aarum et al., 2003). Microglia were later found to secrete mitogenic factors that induce neural stem cell proliferation in a PI3K and Notch-dependent fashion (Morgan et al., 2004), similar to *Drosophila* cortex glia (Pérez-Gómez et al., 2013). Furthermore, addition of microglia can prolong neurogenesis in cultured subventricular zone-derived neurospheres (Walton et al., 2006). Finally, analysis of PU.1^–/–^ mice, which disrupts microglial development, revealed a decrease in cortical progenitor proliferation *in vitro* (Antony et al., 2011). *In vivo* analysis of microglia-dependent embryonic neurogenesis is limited, yet microglia have been shown to regulate the timing of neural differentiation in the zebrafish developing retina (Huang et al., 2012). Here, knockdown of the cell surface protein Colony stimulating factor-1 receptor suppressed entry of microglia into the CNS. Lack of microglia delayed the differentiation of retinal neurons and increased the number of proliferative progenitors, resulting in an immature optic lobe (Huang et al., 2012).

In mammals, both microglia and macroglia regulate adult neurogenesis (reviewed in Falk and Götz, 2017). Co-culturing astrocytes with adult NSCs derived from the hippocampus is sufficient to induce NSC proliferation *in vitro* (Song et al., 2002). Elegant data both *in vitro* and *in vivo* have since defined that complex extrinsic cues from astrocytes and oligodendrocyte precursor cells can bias adult NSCs toward neurogenic or gliogenic trajectories (Peretto et al., 2004; Lie et al., 2005; Barkho et al., 2006; Ferrón et al., 2011; Ashton et al., 2012; Shin et al., 2015). For example, astrocyte-derived Wnt7a instructs neurogenesis (Moreno-Estellés et al., 2012), whereas oligodendrocyte precursor-derived Wnt3a promotes generation of additional oligodendrocyte precursor cells (Ortega et al., 2013). Microglia are primarily thought to regulate hippocampal neurogenesis through pruning of supernumerary neurons that undergo apoptosis (discussed further below, Sierra et al., 2010). Interestingly, a recent report found that microglial phagocytosis induces a cell-autonomous transcriptional cascade, resulting in secretion of factors that are required for continued proliferation of residual neural stem cells. Accordingly, mouse models with disrupted microglial phagocytosis (P2Y12^–/–^ and microglia-specific knockout of MerTK/Axl) showed reduced hippocampal neurogenesis (Diaz-Aparicio et al., 2020). Together, these data show that glia influence developmental and adult neurogenesis through modulation of neural proliferation, and survival.

### Glial Cell Dysfunction and Diseases That Alter Brain Size

Since neurons are postmitotic, neuroepithelial expansion and neural stem cell proliferation are key to regulating neuronal numbers, and consequently, organ size. Defects in neuroepithelial expansion, neural stem cell proliferation, as well as the timing of transitions between these states may therefore lead to developmental disorders that alter brain size (e.g., microcephaly or macrocephaly) (Jamuar and Walsh, 2015). Macrocephaly, characterized by increased head circumference, is also common in autism patients (Lainhart et al., 2006; Grandgeorge et al., 2013; Gamsiz et al., 2015). Indeed, a mammalian model of autism/macrocephaly showed over-proliferation of neural precursors caused by deregulation of mTOR/PI3K signaling (Zhang et al., 2020), which is known to instruct stem cell proliferation during development (Chell and Brand, 2010; Sousa-Nunes et al., 2011; Yuan et al., 2020).

Given that altering the duration of radial glial proliferation can bias cell fate decisions (Pilaz et al., 2016), defects in radial glial proliferation could manifest in many different neurodevelopmental disorders. In humans, mutations in *Fragile X Mental Retardation 1* (*FMR1*) causes Fragile X syndrome, a disease associated with severe cognitive deficits and autism-like behaviors (Salcedo-Arellano et al., 2020). In *Drosophila*, Fmr1/Fmrp is required in neuroblasts and non-autonomously in glia for timely neuroblast reactivation, which is dependent on insulin-like signaling (Callan et al., 2012). Interestingly, FMR1/FMRP is robustly expressed in microglia during brain development (Gholizadeh et al., 2015), and consequently, microglial numbers are reduced in FMR1/FMRP knockout mice (Lee et al., 2019). Since microglia are able to secrete Insulin to induce post-natal neural survival (Ueno et al., 2013), they may also play an important role in neuronal survival or stem cell activation in the etiology of Fragile X syndrome.

The regulation of neurogenesis in mammalian adult neural stem cell niches is especially relevant in the context of neurodegenerative diseases or injury, where the replacement of dead neurons is necessary for recovery (Ghosh, 2019). Adult neurogenic niches are populated by microglia and astrocytes which can modulate cell number and cell fate decisions (Alvarez-Buylla et al., 2008; Sierra et al., 2010; Than-Trong and Bally-Cuif, 2015; Bonzano et al., 2018; Sirerol-Piquer et al., 2019; Vicidomini et al., 2020). NSC reactivation is induced by exercise as well as feeding, which involve insulin signaling fluctuations (Bjornsson et al., 2015; Licht and Keshet, 2015; Otsuki and Brand, 2020). Astrocytic as well as microglial expression of IGF1 has been linked to adult NSC proliferation (Yan et al., 2006; Ueno et al., 2013). These findings raise the possibility that Insulin-resistance associated with Alzheimer’s disease (AD) could influence glial-mediated NSC proliferation, and thus, regeneration (Ferreira et al., 2018). Indeed, AD models show a decrease in neuronal progenitor proliferation (Rodríguez and Verkhratsky, 2011). Additionally, levels of IGF1 receptor as well as insulin are dysregulated in AD animal models in human disease tissue, including in astrocytes (Crews et al., 1992; Connor et al., 1997; Frölich et al., 1999; Jafferali et al., 2000; Steen et al., 2005; Moloney et al., 2010; Gupta et al., 2018). Whether these changes are pathogenic is not yet clear; however, further analysis of the role of glia in regulation of Insulin/IGF1 signaling and NSC proliferation may be an important avenue of investigation to understand AD development and progression.

## Developmental Cell Death and Neuronal Survival

In addition to neuroepithelial expansion and stem cell proliferation, neuronal numbers can also be modulated by programmed cell death (PCD). PCD is essential for appropriate nervous system development. PCD eliminates excess, obsolete, or “damaged/incorrect” cells, and therefore serves as a means to adjust neuronal populations to refine circuit formation (Oppenheim, 1991; Buss et al., 2006; Miura, 2012; Yamaguchi and Miura, 2015). Different types of PCD occur during development, but the most well-studied is apoptosis, which is conserved in invertebrates and vertebrates (Kornbluth and White, 2005; Maghsoudi et al., 2012; Zakerp et al., 2015). When triggered, apoptosis induces a signaling cascade that ultimately results in activation of caspases that completely break down the proteome of the cell, priming the cell for elimination (Elmore, 2007; Singh et al., 2019). The decision to trigger PCD is controlled by a balance of cell death and cell survival signals, and glia are active participants in this regulatory process (Burek and Oppenheim, 1996; Frade and Barde, 1998; Sedel et al., 2004; Bredesen et al., 2006; Coutinho-Budd et al., 2017).

### Regulation of Programmed Cell Death by Glia

One of the earliest studies to define glia-derived signals that activate neuronal death was described in the chick embryonic retina. This pioneering work demonstrated that the secreted neurotrophic factor NGF (Nerve Growth Factor) is exclusively expressed by microglia during early embryogenesis and is required to induce cell death in the early retinal neuroepithelium (Frade and Barde, 1998). NGF can promote cell survival through binding and activation of receptor tyrosine kinases (TrkA); however, here, NGF leads to cell death through neurotrophin p75 receptors (Frade and Barde, 1998). Cell death is prevalent in this epithelium in vertebrates and is essential for normal development (Vecino et al., 2004), although the exact physiological role of this high death rate is not known. It is hypothesized that PCD may be necessary to create space for the incoming axons of retinal ganglion cells forming the optic nerve at the center of the retina (Frade and Barde, 1998).

Microglia play a similarly critical role in controlling cell death within the developing motor circuit. Motoneurons are born in excess in the vertebrate spinal cord (Hamburger and Oppenheim, 1982; Pettmann and Henderson, 1998; Eisen and Melancon, 2001; Sedel et al., 2004). During late embryonic development, post-mitotic motoneurons compete for trophic factors during synaptogenesis and undergo apoptosis in order to reach a 1:1 stoichiometry of motoneuron to myotube. Selective apoptosis is triggered by activation of TNF receptor 1 on motoneurons by the pro-death factor TNFα (Tumor necrosis factor α) secreted by microglia (Sedel et al., 2004). Interestingly, microglia utilize different pro-apoptotic pathways depending on the cellular and developmental context. Integrin CD11b and immunoreceptor DAP12 expressed in microglia similarly promote cell death of hippocampal neurons perinatally (Wakselman et al., 2008). During post-natal development, microglia trigger cell death of Purkinje cells, a cerebellum neural population, through release of superoxide ions (Marín-Teva et al., 2004). Interestingly, microglia can also shape brain development through active engulfment of viable neural precursor cells and oligodendrocyte precursor cells (Cunningham et al., 2013; Nemes-Baran et al., 2020), demonstrating that microglia-mediated engulfment can profoundly impact cell numbers within the CNS.

Glia also regulate neuronal cell numbers by actively promoting cell survival. In *Drosophila*, neuronal cell bodies are surrounded by cortex glia that extend processes to envelop differentiated neurons. During development, correct morphology and physiology of cortex glia is required for the survival of new-born neurons (Coutinho-Budd et al., 2017; Spéder and Brand, 2018; Plazaola-Sasieta et al., 2019; Yuan et al., 2020). Cortex glia exclusively surround neuronal cell bodies. Morphologically, cortex glia are most akin to mammalian satellite glial cells, which have also been shown to promote survival of postganglionic sympathetic neurons in the peripheral nervous system (PNS) (Enes et al., 2020). Further mechanistic and descriptive study of each of these cell types is needed before concluding the functional equivalency of cortex and satellite glia. In the CNS, microglia promote neuronal survival *in vitro* and *in vivo* (Polazzi et al., 2001; Ueno et al., 2013; Littlejohn et al., 2020). Specifically, microglial-derived IGF1 can promote survival of layer V cortical neurons during post-natal development (Ueno et al., 2013) and in new-born hippocampal neurons after injury (Littlejohn et al., 2020). The balance between apoptosis and neuronal survival during nervous system development is essential to promote maturation and homeostasis of newly formed neuronal circuits (Marín-Teva et al., 2004; Pfisterer and Khodosevich, 2017); thus, glia are key mediators of circuit development (discussed further below).

### Clearance of Neuronal Debris by Glia in Development

In addition to regulating overall cell numbers, glia modulate brain development through clearance of neural debris following apoptosis (Marín-Teva et al., 2004) and during activity-dependent neuronal remodeling (Fuentes-Medel et al., 2009; Paolicelli et al., 2011; Santosa et al., 2018; Wilton et al., 2019). Since the CNS is partially isolated from blood circulation by the blood-brain barrier and is therefore inaccessible to circulating macrophages, glial cells with phagocytic capacity are responsible for clearing cellular and axonal debris in the developing and mature nervous system (Kurant, 2011; Shklyar et al., 2014; Shklover et al., 2015; Galloway et al., 2019). As the resident immune cells of the nervous system, this function is performed primarily by microglia in vertebrates (Ferrer et al., 1990; Calderó et al., 2009; Napoli and Neumann, 2009; Cunningham et al., 2013; Lyons and Talbot, 2015; Morsch et al., 2015; Prinz et al., 2019); however, other glial subtypes exhibit phagocytic activity, such as astrocytes (Chung et al., 2013; Iram et al., 2016), olfactory ensheathing cells in the olfactory system (Nazareth et al., 2015), satellite glia precursors of the developing dorsal root ganglia (Wu et al., 2009), Müller glia in the retina (reviewed in Bejarano-Escobar et al., 2017), and Schwann cells that occupy peripheral nerves and neuromuscular junctions (Reichert et al., 1994; Lutz et al., 2017; Santosa et al., 2018). Since there is no microglia equivalent in *Drosophila*, other subtypes of glia take up this role (Figure 1), including cortex glia (Kurant et al., 2008; Doherty et al., 2009; McLaughlin et al., 2019), astrocytes (Tasdemir-Yilmaz and Freeman, 2014; Hilu-Dadia et al., 2018), and ensheathing glia (Doherty et al., 2009; Musashe et al., 2016; Hilu-Dadia et al., 2018). One of the best-described conserved mechanisms for phagocytosis by macroglia is through the engulfment receptor MEGF10 (Multiple EGF-like domains 10), ortholog of Draper in *Drosophila*, and CED-1 in *C. elegans*. This receptor was first shown to be required for phagocytosis by neighboring engulfing cells during *C. elegans* development (Zhou et al., 2001), and soon after, shown to be used for phagocytosis by glial cells in *Drosophila* (Freeman et al., 2003; Awasaki et al., 2006; MacDonald et al., 2006). We now know that mammalian glia require this receptor for both axonal (Chung et al., 2013) and apoptotic cellular debris clearance (Wu et al., 2009; Iram et al., 2016), nicely illustrating the conservation of glial phagocytosis at the molecular and cellular level.

### Ectopic Phagocytic Activity of Glia in Disease

Glial clearance of neural debris is particularly relevant in the context of neurodegenerative diseases, such as Parkinson’s and AD. Phagocytic glial cells seem to have a neuroprotective effect in clearance of toxic protein aggregates characteristic of early stages of degeneration (Yeh et al., 2016; Ho, 2019). However long-term accumulation of aggregates leads to increased microglial and astrocytic activation, and therefore excessive phagocytic activity and inflammation (Glass et al., 2010; Liddelow et al., 2017), creating a chronic toxic environment that aggravates neurodegeneration (discussed further in section “Glial Regulation of Synapse Numbers”). Here, we will focus on glial roles in the modulation of PCD and neuronal survival during nervous system development. Considering the functions of glia in differentially promoting neuronal survival and death during development, disruption of glia-mediated neuronal apoptosis could contribute to the etiology of neurodevelopmental diseases. In fact, recent work in a zebrafish model of RNAseT2-deficient leukoencephalopathy (characterized by myelin defects that lead to motor and cognitive decline) suggest that early developmental defects in apoptotic cell phagocytosis by microglia contribute to pathology (Hamilton et al., 2020). Increases in glia-mediated neuronal apoptosis are similarly detrimental to brain function. Alexander disease is caused by mutations in the glial fibrillary acidic protein (GFAP). Disease-associated mutations result in severe astrocyte dysfunction, causing a leukoencephalopathy that is frequently accompanied by macrocephaly, seizures, and psychomotor retardation (Brenner et al., 2001; Quinlan et al., 2007). Using a *Drosophila* model of Alexander disease, Wang and colleagues recently showed that diseased glia non-autonomously induce neuronal cell death by releasing nitric oxide (Wang L. et al., 2015). Thus, glia non-autonomously regulate cell numbers in development, and loss of this regulation can manifest in human disease.

## Glia in Neuronal Migration and Pathfinding of Axons and Dendrites

From invertebrates to vertebrates, neurons and their progenitors migrate to precise positions within the nervous system, where they will eventually extend axons to find their respective partners. The spatial and temporal precision of neuronal positioning and neurite extension is essential for the assembly of correct neural circuits and for correct nervous system organization (Buchsbaum and Cappello, 2019). Glial cells also migrate to specific positions in the nervous system after being born (Klämbt, 2009), where they act as scaffolds for neuronal migration, and modify the environment to guide neuronal migration and subsequent neurite outgrowth (Allen and Lyons, 2018; Song and Dityatev, 2018; Rigby et al., 2020).

### Glia and Neuronal Migration

Two major types of neuronal migration occur during the development of the mammalian cortex. Radial migration describes the migration of new-born neurons from the ventricular zone (VZ), where they originate, to the most superficial cortex layer (marginal zone). This pattern of migration results in an inside-out organization, with early born cortical neurons in superficial layers and later-born neurons in deep layers. While excitatory neurons migrate radially, inhibitory interneurons populate the cortex by tangential migration from the ganglionic eminences to the cortex, slotting horizontally into the cortical layers (Moon and Wynshaw-Boris, 2013; Buchsbaum and Cappello, 2019). As mentioned above, radial glia are the neural progenitors of the vertebrate CNS (Doetsch et al., 1999; Noctor et al., 2001), but they also serve as scaffolds for radial migration. Radial glia extend processes spanning from the VZ to the surface of the neural tube (pial surface), and these processes are substrates for neuronal migration (Rakic, 1988; Marín and Rubenstein, 2003). Interestingly, radial glia also regulate the positioning of Cajal–Retzius cells (Kwon et al., 2011), which migrate tangentially to populate the marginal zone and are essential for the correct layering of the developing cortex (reviewed in Frotscher, 1998). Microglia, in contrast to macroglia, are also present in the CNS during migration of cortical neurons (Thion and Garel, 2017). Thus, microglia are present at the right time and place to regulate cortical migration. Indeed, embryonic depletion or ectopic activation of microglia results in mispositioning of *Lhx6+* interneurons (tangentially migrating) to upper cortical layers during early postnatal development (Squarzoni et al., 2014), showing an important role of microglia in the organization of brain circuits.

In the peripheral nervous system, glial cells also contribute to neuronal migration. Within the olfactory system, Gonadotropin-releasing hormone (GnRH) neurons originate from the olfactory placode. GnRH neurons then migrate along the olfactory sensory neuron axons encompassed by the olfactory ensheathing cells into the developing forebrain (Wray, 2010). In the absence of *Sox10* (required for neural crest-derived glial cells), olfactory ensheathing cells do not develop and GnRH neurons fail to enter the forebrain, resulting in accumulation of GnRH neurons at the PNS-CNS boundary. Additionally, loss of olfactory ensheathing cells disrupts GnRH axon targeting, demonstrating a dual role for these glial cells in organization of the neural circuits underlying olfaction (Barraud et al., 2013).

### Glial Regulation of Neurite Outgrowth

Glial cells within the PNS and CNS are essential for proper guidance of axon and dendrite outgrowth (Mason and Sretavan, 1997; Poeck et al., 2001; Hidalgo, 2003; Chotard and Salecker, 2004; Chotard et al., 2005; Learte and Hidalgo, 2007; Procko and Shaham, 2010; Avraham et al., 2020; Rigby et al., 2020). This is required for directing information flow in local circuits and is also essential for broad nervous system organization. The role of glia in axon guidance has been particularly well-defined in invertebrate species. Following initial pathfinding by pioneer axons, embryonic longitudinal glia form a scaffold that enables assembly of the longitudinal connectives in the *Drosophila* ventral nerve cord, analogous to the vertebrate spinal cord (Hidalgo et al., 1995; Hidalgo and Booth, 2000). This function is primarily performed by cortex glia and neuropil glia in larvae to accommodate for second waves of neurogenesis (Spindler et al., 2009). In the embryo, ablation of longitudinal glia or pioneer axons results in misrouting of secondary axons from interneurons. Consequently, longitudinal connectives fail to form due to defasciculation (Hidalgo et al., 1995; Hidalgo and Booth, 2000). Similarly, loss of Schwann cells does not impair initial outgrowth of axons in the peripheral lateral line nerve in zebrafish, but results in progressive defasciculation (Raphael et al., 2010; Perlin et al., 2011).

Beyond longitudinal growth, glia also serve as “guidepost cells” to ensure proper guidance of axons that must cross the midline in Bilateria species (reviewed in Freeman and Doherty, 2006; Chotard and Salecker, 2007; Squarzoni et al., 2015). In the *Drosophila* embryo, a specialized population of glial cells, aptly named “midline glia,” migrate and position themselves at the CNS midline. At the midline, they ensheath the axons that compose the longitudinal tracts and commissures, and express highly conserved guidance cues that direct axonal crossing (e.g., Netrin and Slit) (Klämbt et al., 1991; Jacobs, 2000; Lemke, 2001; Learte and Hidalgo, 2007; Stork et al., 2009; Crews, 2010; Howard et al., 2019). Vertebrates have a similar population of cells that populate the ventral midline of the developing spinal cord, floorplate cells, which also use Netrins and Slit as attractive and repellent cues to regulate crossing of commissural axons, respectively (reviewed in Chotard and Salecker, 2004; Placzek and Briscoe, 2005; Crews, 2010; Evans and Bashaw, 2010). In vertebrates, communication via commissures between the two brain hemispheres can also be regulated by glia. In zebrafish, astrocyte-like glia form glial bridges across the midline of the forebrain and secrete Slit1a/Slit2/Slit3 to guide commissural and retinal axons through the anterior and postoptic commissures (Barresi et al., 2005). Correct axon guidance across the anterior commissure also requires expression of *Slit2* from astrocytes in mice (Minocha et al., 2015). Moreover, both wedge glia and microglia are required for correct guidance and fasciculation of axons through the developing corpus callosum (Shu et al., 2003; Pont-Lezica et al., 2014). In general, loss of these conserved glial-derived cues results in defasciculation of commissural axons, and to a lesser extent, failed midline crossing (Hidalgo et al., 1995; Hidalgo and Booth, 2000; Shu et al., 2003; Barresi et al., 2005; Minocha et al., 2015).

Interestingly, recent work in *C. elegans* has defined a glial cell population that instructs the initial assembly of the central nerve ring. The cephalic sheath (CEPsh) glia, which envelops the outer surface of the nerve ring once formed, extend processes to demarcate the nerve ring prior to arrival of any axonal projections. Once there, a bundle of fewer than 10 pioneer axons enter the nerve ring, followed by a stereotyped pattern of follower axons. By taking advantage of forward genetic screening approaches, it was shown that glial-derived Netrin and Semaphorin instruct pathfinding of pioneer and follower axons into the nascent nerve ring, respectively (Yoshimura et al., 2008; Rapti et al., 2017). Thus, glia are essential directors of neurite outgrowth at local and global scales.

### Glial Regulation of Transition Zones

Glial cells also guide information flow by establishing boundaries throughout the nervous system, including within the CNS and PNS to separate functional units (Wandall et al., 2005; Chotard and Salecker, 2007; Kucenas, 2015; Wu et al., 2017; Bittern et al., 2020), and at the border between the CNS and PNS. Here, we focus on the role of glia at unique borders called transition zones. Transition zones are the specialized regions in the nervous system where the CNS and PNS meet (Fontenas and Kucenas, 2017; Suter and Jaworski, 2019). Because glial cell populations traverse the CNS:PNS interface in development (Sepp et al., 2001; Kucenas et al., 2008, 2009; Clark et al., 2014; Smith et al., 2014, 2016) and following injury (Adelman and Aronson, 1972; Blakemore and Patterson, 1975; Franklin and Blakemore, 1993; Sims et al., 1998; Green et al., 2019), this boundary must be selectively permeable. Accordingly, there are glial cell populations that sit at transition zones and regulate exit and entry of neurite projections and in some cases, whole cells (Fontenas and Kucenas, 2017). In the zebrafish spinal cord, CNS-derived glial cells populate the motor exit point (MEP) and myelinate the dorsal-most segments of motor axons. Additionally, MEP glia act as gatekeepers to prevent ectopic exit of oligodendrocytes from the spinal cord (Smith et al., 2014; Fontenas et al., 2019). In mammals and chicks, neural crest-derived boundary cap cells occupy the dorsal root exit zone and the MEP transition zone (Niederländer and Lumsden, 1996; Golding and Cohen, 1997; Vermeren et al., 2003). Cre-mediated (Krox20+) knockout of *Sox10* does not disrupt boundary cap cell formation but results in migration of oligodendrocytes and astrocytes into the periphery (Fröb et al., 2012). In addition, boundary cap cells prevent ectopic exit of motor neuron cell bodies from the CNS (Vermeren et al., 2003; Bron et al., 2007). More investigation of mammalian transition zones will uncover whether neural crest-derived MEP glia are also present, and if so, how they interface with boundary cap cells. Nonetheless, despite their distinct origins, MEP glia and boundary cap cells share functional similarities and both express *Wnt-inhibitory factor 1* (Coulpier et al., 2009; Smith et al., 2014; Fontenas and Kucenas, 2018), implicating *Wnt-inhibitory factor 1* as a potential key regulator of transition zone function. *Drosophila* also have glial cell populations that occupy transition zones in the ventral nerve cord (Sepp et al., 2000, 2001) and in the central brain (Chotard and Salecker, 2007). In the developing eye, photoreceptors in the retina differentiate and extend their axons through the optic stalk in order to project into the optic lobe, and glial cells within the retina are necessary for this PNS to CNS transition (Rangarajan et al., 1999). A similar phenomenon is observed in the mammalian olfactory system (Barraud et al., 2013), as discussed above. In sum, glia are instrumental in guiding nervous system organization.

### Glial Contribution to Diseases Impacting Nervous System Organization

Glia are critical regulators of axon targeting during development (Mason and Sretavan, 1997; Hidalgo, 2003; Chotard and Salecker, 2004; Learte and Hidalgo, 2007; Procko and Shaham, 2010; Rigby et al., 2020) and during regeneration (Doherty et al., 2014; Lewis and Kucenas, 2014; Jessen and Mirsky, 2016; Purice et al., 2016; Sofroniew, 2018). Accordingly, changes to glial signaling can profoundly impact axon regrowth after injury. As this topic has been recently reviewed (Rigby et al., 2020); here, we will focus on how defective glial signaling during neuronal migration contributes to human disease. Neural migration disorders (NMDs) are characterized by defects in cortical neuron migration that produce a spectrum of phenotypes including intellectual disability, epilepsy, developmental delay, and motor impairment (Buchsbaum and Cappello, 2019). The classic NMD is Lissencephaly type I, or “smooth brain,” characterized by the lack of folds (gyri) on the cerebral surface and an inverted organization of the cortical layers (Moon and Wynshaw-Boris, 2013). Because cerebral gyri are not present in murine models, recent efforts to understand the etiology of NMDs have expanded beyond the mouse to alternative model systems such as non-human primates, ferrets, and human cerebral organoids derived from patient tissue (Wynshaw-Boris et al., 2010; Bershteyn et al., 2017; Wang, 2018; Pinson et al., 2019). These works indicate that defects in radial glia proliferation strongly contribute to reduced brain size in lissencephaly patients (Gambello et al., 2003; Pramparo et al., 2010; Wynshaw-Boris et al., 2010; Bershteyn et al., 2017); however, defects in the radial glial scaffold also contribute to lissencephaly (Caviness, 1982; Trommsdorff et al., 1999; Dulabon et al., 2000; Li et al., 2008). Mutations in *REELIN*, which encodes a glycoprotein, cause an autosomal recessive form of lissencephaly. *Reeler* mice (mutant for *Reelin*) exhibit over migration of neurons on radial glial processes and inverted layering of the mammalian cortex (Caviness, 1982; Trommsdorff et al., 1999; Dulabon et al., 2000). Similar to the neurons they support, astrocytes exhibit layer specific expression profiles in the cortex, which is also inverted in *Reeler* mice (Bayraktar et al., 2020). Astrocytes are the primary synapse-associated glial cell type and are essential for maintenance of synapse health and function (see below in section “Glial Regulation of Circuit Function”). Because astrocytes represent a heterogeneous population of cells with brain region and circuit level specificity (Bayraktar et al., 2014, 2020; Molofsky et al., 2014; John Lin et al., 2017), disorganization of astrocytes could contribute to the development of seizures in lissencephalic patients.

Finally, as discussed above, microglia arrive in the CNS concurrent with neurogenesis. Microglia have been shown to regulate tangential migration (Squarzoni et al., 2014). Although lissencephaly is primarily thought to originate from defects in radial migration, defects in tangential migration are also apparent in mouse models and human patients of Lissencephaly type I (McManus et al., 2004; Pancoast et al., 2005; Kappeler et al., 2006, 2007; Friocourt et al., 2007; Marcorelles et al., 2010). Therefore, this glial subtype is of special interest in the study of diseases with early migratory defects, including cortical malformations and other neurodevelopmental diseases with targeting defects (Squarzoni et al., 2015).

## Glial Regulation of Synapse Numbers

Following initial waves of axonal and dendrite growth, developing neurons enter a phase of rapid synaptogenesis. Glia shape overall synapse numbers within the nervous system through three primary means: (1) regulation of synapse development, (2) selective pruning of supernumerary connections, and (3) continued synapse maintenance (discussed in section “Glial Regulation of Circuit Function”).

### Glial Regulation of Synaptogenesis

The first evidence that glia instruct synaptic development stemmed from studies of cultured retinal ganglion cells (RGCs) (Pfrieger and Barres, 1997; Ullian et al., 2001). In these pioneering works, RGCs cultured in isolation exhibited an intrinsic ability to form a limited number of synapses, which was significantly enhanced when grown in astrocyte-conditioned medium (Ullian et al., 2001). More recently, astrocyte-like CEPsh neuropil glia in *C. elegans* (Colón-Ramos et al., 2007; Shao et al., 2013) and *Drosophila* astrocytes (Muthukumar et al., 2014) were similarly shown to instruct synaptogenesis. Indeed, TEM analysis revealed that ablation of *Drosophila* astrocytes results in a 30–50% reduction in global synapse numbers in young adult flies, depending on brain region (Muthukumar et al., 2014). In the last decade, a series of astrocyte-secreted molecules (Hevin, Thrombospondins, Glypicans, Cholesterol, and more) were identified as essential for development of structural and functional synapses in mammals and invertebrates (Allen and Eroglu, 2017). As this subject has been extensively reviewed (Chung et al., 2015; Allen and Eroglu, 2017; Farhy-Tselnicker and Allen, 2018), we will instead focus on the role of additional glial cell types in synaptogenesis.

As aforementioned, microglia are known to regulate both neurogenesis and axon targeting (Pont-Lezica et al., 2014; Ueno and Yamashita, 2014). Microglia represent the resident immune cells of the brain and are well known for their roles in synapse engulfment (see more in *Synaptic pruning*). Interestingly, depletion of microglia during early postnatal development, a period of rapid synaptogenesis, results in a significant reduction in spine density on Layer 2/3 pyramidal neurons in mouse (Miyamoto et al., 2016). Further, live imaging demonstrated that microglial-dendritic contact was sufficient to induce spine formation (Miyamoto et al., 2016). These findings suggest that microglia also promote synapse development. Accordingly, microglia have been shown to regulate learning-dependent synapse formation in the adult brain. Motor skill learning stimulates formation of dendritic spines within the motor cortex to facilitate improvement of motor performance (Yang et al., 2009; Liston et al., 2013). When microglia are depleted in postnatal mice (P19-P30), learning induced spine formation on layer V pyramidal neurons is impaired. Microglia-specific deletion of Brain-derived neurotrophic factor (BDNF) recapitulates this phenotype, suggesting that microglial BDNF drives learning-induced post-synaptic dendritic spines within the motor cortex (Parkhurst et al., 2013), but whether BDNF regulates synapse development more broadly was not assessed. Furthermore, a recent report found that environmental enrichment induces robust expression of IL-33 in a subset of hippocampal neurons, which stimulates microglia-dependent spine formation. Accordingly, conditional knockout (cKO) of IL-33 from neurons in the dentate gyrus and CA1 regions, or cKO of the obligate co-receptor *Il1rl1* (*Interleukin 1 receptor like 1*) from microglia, suppressed experience-dependent spine formation (Nguyen et al., 2020). Excitingly, this study found that IL-33 expression stimulates microglia-dependent engulfment of Aggrecan (Nguyen et al., 2020), a chondroitin sulfate proteoglycan that forms part of the perineuronal nets that restrict neuronal plasticity (Rowlands et al., 2018). Expression of chondroitin sulfate proteoglycans suppresses activity-dependent neural circuit remodeling during early developmental windows called critical periods (Takesian and Hensch, 2013; Rowlands et al., 2018). It will be important to determine whether microglia also promote synapse formation during early phases of development by maintaining the extracellular matrix in an environment that is permissive to synaptogenesis.

Outside of the CNS, peripheral glia are also active participants in synaptogenesis at the neuromuscular junction (NMJ). In *Drosophila*, NMJ glia secrete the TGF-β ligand Maverick in concert with muscle-derived TGF-β ligands (Glass bottom boat), which activate TGF-β receptors on motor axons to stimulate synaptic growth (Fuentes-Medel et al., 2012). Similarly, terminal Schwann cells (tSCs) at the vertebrate NMJ are necessary for expansion of motor endplates during development (Love and Thompson, 1998; Herrera et al., 2000; Darabid et al., 2014; Santosa et al., 2018), and stimulate reinnervation and synapse development following injury (Kang et al., 2003, 2019; Darabid et al., 2014; Santosa et al., 2018; Jablonka-Shariff et al., 2020). For example, extending the time to reinnervation following more severe cut injury of the nerve innervating the sternomastoid or soleus muscle in young adult mice (2–4 months post-natal) results in migration of tSCs away from the NMJ. Because motor axons depend on tSC guidance to the appropriate targets during regrowth, reinnervation to NMJs was misrouted and significantly delayed where tSCs were absent (Kang et al., 2014). Although the molecular mechanisms by which tSCs promote synaptogenesis following injury are limited, a recent report found tSCs lacking expression of the adhesion G protein-coupled receptor Gpr126/Adgrg6 fail to extend processes to guide reinnervation following crush injury of the sternomastoid muscle (Jablonka-Shariff et al., 2020). As 35% of the drug targets on the market currently target G protein-coupled receptors (Sriram and Insel, 2018), this study implicates Gpr126 (Diamantopoulou et al., 2019) as a potential therapeutic target to promote axon regrowth and synapse development following human peripheral nerve injuries.

### Glia-Mediated Synaptic Pruning

During development, synapses are often generated in excess of what is necessary to achieve functional neural circuits. Accordingly, circuits undergo waves of activity-dependent circuit refinement to prune away weak or redundant synapses, followed by strengthening and maintenance of surviving synapses (Riccomagno and Kolodkin, 2015; Jung et al., 2019). As noted previously (see section on “Developmental Cell Death and Neuronal Survival”), one major pruning pathway used by both invertebrate and vertebrate glia is the CED-1/Draper/Megf10 pathway (Zhou et al., 2001; Freeman et al., 2003; Awasaki et al., 2006; MacDonald et al., 2006; Tasdemir-Yilmaz and Freeman, 2014; Chung et al., 2015). Loss of this pathway from mammalian astrocytes and microglia impairs activity-dependent pruning of excess RGC synapses in the developing visual system (Chung et al., 2013). Similarly, *Drosophila* NMJ glia use Draper to phagocytose excess synaptic boutons on motor axons during NMJ development (Fuentes-Medel et al., 2009).

Although astrocytes contribute to synaptic pruning and remodeling (Chung et al., 2013; Hill et al., 2019), the primary phagocytic cells within the vertebrate CNS are microglia (Paolicelli et al., 2011; Wilton et al., 2019). As immune cells, microglia primarily respond to immune cues (e.g., cytokines) to guide their behaviors (Werneburg et al., 2017). The best described signaling axis that drives microglial-dependent synaptic pruning is the complement pathway (Hammond et al., 2018). This pathway has been extensively studied in both developmental (Stevens et al., 2007; Schafer et al., 2012; Filipello et al., 2018) and pathological conditions (Shi et al., 2015; Hong et al., 2016; Schafer et al., 2016; Vasek et al., 2016; Werneburg et al., 2020). In brief, during early postnatal development, synapses that are destined for elimination secrete the complement components C1q and C3 as “eat me” signals that bind to the fractalkine receptor CX3CR1 on microglial membranes to trigger engulfment of the tagged synapse (Hammond et al., 2018). Microglia also receive “don’t eat me” signals (e.g., CD47) to prevent ectopic synapse pruning (Lehrman et al., 2018). Interestingly, TGF-β signaling from astrocytes can stimulate C1q expression in RGCs to potentiate microglial engulfment (Bialas and Stevens, 2013). Furthermore, astrocyte-derived IL-33 can bind to IL1RL1 on microglia to stimulate engulfment of excitatory glutamatergic synapses in the thalamus, and to instruct engulfment of both excitatory and inhibitory inputs onto α motor neurons in the spinal cord (Vainchtein et al., 2018). These studies suggest that astrocytes can greatly influence circuit refinement not only through direct synapse engulfment (Chung et al., 2013), but by directing microglia-dependent pruning (Bialas and Stevens, 2013; Vainchtein et al., 2018). Although a number of neurons, microglia, and astrocyte-derived proteins have been implicated in microglial dependent pruning, how synapses are physically tagged for removal remained elusive until recently. Phosphatidylserine is a phospholipid within cell membranes that is usually localized to the inner leaflet of the plasma membrane but can be externalized to mark cells for apoptosis (Segawa and Nagata, 2015). A new study found that exposed phosphatidylserine within pre-synaptic membranes on hippocampal neurons binds to C1q and serves as an “eat me” signal, which in turn promotes microglial engulfment through the phagocytic receptor TREM2 (Filipello et al., 2018; Scott-Hewitt et al., 2020). Interestingly, a recent study found that TWEAK-expressing microglia drive synapse elimination in the developing visual system independent of their role in phagocytosis (Cheadle et al., 2020). Thus, glia instruct synapse numbers by promoting both developmental and activity-dependent synaptogenesis, followed by pruning (phagocytic and non-phagocytic) of excess synapses to promote circuit refinement.

### Glia-Mediated Changes to Synapse Stability in Disease

Defects in a number of the aforementioned signaling pathways have been linked to both neurodevelopmental and neurodegenerative disorders (Hong et al., 2016; Vasek et al., 2016; Allen and Eroglu, 2017; Werneburg et al., 2020). Misexpression of astrocyte-derived synaptogenesis genes (e.g., Glypicans 4 and 6, Hevin, Thrombospondin 1) is evident in several autism spectrum disorders, suggesting that astrocyte dysfunction may strongly influence developmental circuit defects in humans (Allen and Eroglu, 2017). Additionally, mutant astrocytes (Lioy et al., 2011) and microglia (Schafer et al., 2016) both contribute to circuit dysfunction in a mouse model of Rett’s syndrome, a neurodevelopmental disorder characterized by cognitive and motor deficits. Finally, ectopic expression of synapse pruning pathways is evident in animal and human models of neurodegeneration. Complement-driven phagocytosis of synapses by microglia is present in preclinical mouse models of multiple sclerosis (Werneburg et al., 2020), Huntington’s disease (Hong et al., 2016), and prior to viral-induced memory impairment (Vasek et al., 2016). Furthermore, allelic variations of complement protein C4 are linked to increased susceptibility to schizophrenia in humans (Sekar et al., 2016; Kamitaki et al., 2020). These data implicate ectopic microglial pruning as a common underlying factor that contributes to, and may even instigate, circuit dysfunction in neurological disorders (Werneburg et al., 2017; Hammond et al., 2018).

## Glial Regulation of Synaptic Maintenance and Basic Synaptic Function

### Ion Buffering by Glia

Following synapse establishment and refinement, perisynaptic glia ensure a permissive local environment that is absolutely essential for regulating synapse health, maintenance, and function. Proper synaptic transmission is dependent on efficient depolarization and repolarization of neuronal membranes in response to stimuli. This is achieved through tight regulation of the ionic milieu surrounding synapses (Na^+^, K^+^, and Cl^–^), which is highly dependent on glia (Featherstone, 2011; Leiserson and Keshishian, 2011; Khakh and Sofroniew, 2015; Olsen et al., 2015; Parinejad et al., 2016). Within CNS gray matter, astrocytes are the predominant glial cell population that maintains ion homeostasis around synapses (Olsen et al., 2015; Ohno, 2018). Astrocytes express a myriad of ion channels and transporters (Olsen et al., 2006, 2015; Seifert et al., 2009, 2018; Zhang et al., 2014), but are best known for their role in potassium buffering via Kir4.1 (Kucheryavykh et al., 2007; Olsen et al., 2015). Kir4.1 is a glia-specific, inwardly rectifying K^+^ channel that facilitates perisynaptic K^+^ homeostasis, but also contributes to astrocyte glutamate uptake and overall astrocyte health (Dibaj et al., 2007; Djukic et al., 2007). Accordingly, astrocyte-specific knockout of Kir4.1 in mice disrupts astrocyte morphogenesis, significantly impairs K^+^ and glutamate uptake, and results in a decrease in spontaneous network activity in the CA1 stratum radiatum (Djukic et al., 2007). In CNS white matter, astrocytic processes are only able to contact neurons at the nodes of Ranvier (Wang et al., 1993; Rash et al., 2016; Larson et al., 2018), necessitating additional glial populations for efficient K^+^ buffering. Both oligodendrocyte progenitor cells and myelinating oligodendrocytes strongly express Kir4.1 (Kalsi et al., 2004; Zhang et al., 2014), and contribute to clearance of K+ from heavily myelinated regions of the brain that are inaccessible to astrocytic processes (Larson et al., 2018). Together, astrocytes and oligodendrocytes mediate ion homeostasis across gray and white matter regions of the vertebrate CNS, respectively, which is essential to both synaptic and circuit level function (discussed below in section “Glial Contribute to Circuit Function”).

Peripheral glia buffer extracellular ion concentrations to promote long-term synaptic and axonal health. In *C. elegans*, the K^+^/Cl^–^ cation-chloride cotransporter KCC-3 is strongly localized to membranes of the amphid sheath (AMsh) glia that support neuron receptive endings on thermosensing AFD neurons. Mutations in *kcc-3* result in progressive retraction of neuron receptive endings (Singhvi et al., 2016). Furthermore, *kcc-3* mutants fail to elicit sustained temperature-induced activation of AFD neurons, likely as a result of depleted extracellular K^+^ (Yoshida et al., 2016). During mouse cochlear development, activation of the purinergic autoreceptor P2RY1 on glia-like inner supporting cells triggers release of Cl^–^ through TMEM16A Ca2^+^-activated Cl^–^ channels and subsequent release of K^+^ ions to maintain charge. This series of ion exchanges causes spontaneous depolarization of inner hair cells to initiate maturation of auditory neurons prior to the onset of hearing (Wang H. C. et al., 2015; Babola et al., 2020). Thus, glia-mediated ion buffering is essential during critical periods of circuit development and is necessary for long term synaptic health.

### Glial Regulation of Neurotransmitter Turnover

In addition to ion homeostasis, vertebrate and invertebrate glia express a suite of receptors and transporters for uptake of neurotransmitters including glutamate, acetylcholine, and GABA, as well as neuromodulators such as histamine (van der Zee et al., 1993; Ribak et al., 1996; Stacey et al., 2010; Schousboe et al., 2013; Allen, 2014; Chaturvedi et al., 2014; Muthukumar et al., 2014; Boddum et al., 2016; Katz et al., 2019; Peng et al., 2019; Singhvi and Shaham, 2019; Zhang et al., 2019). Continual recycling of neurotransmitters by perisynaptic glia, primarily astrocytes, is necessary for prolonged synaptic function. Neurotransmitter uptake serves to prevent ectopic crosstalk between neighboring synapses, but also to avert excitotoxicity due to accumulation of perisynaptic glutamate (Murphy-Royal et al., 2017). Once taken up by astrocytes, glutamate enters the glutamate-glutamine cycle. In this cycle, glutamine synthase converts glutamate into glutamine for subsequent release and uptake by neurons. Because glutamine is a metabolic precursor for both glutamate and GABA, this is essential for continued function of the predominant excitatory and inhibitory synapse types within the brain (Schousboe et al., 2013; Allen, 2014). Interestingly, a recent report found that oligodendrocytes within caudal regions of the brain and spinal cord also express glutamine synthetase. Following oligodendrocyte-specific knockout of glutamine synthetase, synaptic glutamate transmission in the midbrain was significantly impaired due to overall reductions in both glutamate and glutamine (Xin et al., 2019). Thus, in the CNS, both oligodendrocytes and astrocytes mediate continued turnover of glutamate.

### Glial-Mediated Synaptic Dysfunction in Disease

Loss of ionic and neurotransmitter homeostasis disrupts synaptic maintenance and synaptic function, and has consequently been linked to a number of neurological disorders. Mutations in *KCNJ10*, which encodes human Kir4.1, causes SeSAME/EAST syndrome. SeSAME/EAST is a neurodevelopmental disorder associated with a variety of phenotypes including ataxia, seizures, deafness, and intellectual disability (Scholl et al., 2009). Furthermore, mutations in *KCNJ10* are linked to increased risk for seizure and ataxia in other neurodevelopmental disorders such as autism and Rett’s syndrome, underscoring the importance of glial ion buffering to developmental circuit function (Nwaobi et al., 2016; Eid et al., 2019). Interestingly, though mutations in *KCNJ10* have not been linked to neurodegenerative disorders, analysis of animal models of AD, amyotrophic lateral sclerosis (ALS), and Huntington’s disease suggest that downregulation of Kir4.1 in astrocytes may contribute to disease onset and/or progression (Kaiser et al., 2006; Wilcock et al., 2009; Tong et al., 2014). Indeed, post-mortem brain samples from AD patients with severe cerebral amyloid angiopathy showed loss of Kir4.1 from astrocytic endfeet (Wilcock et al., 2009). Altered neurotransmitter turnover is also linked to neurodevelopmental and neurodegenerative disorders (Murphy-Royal et al., 2017), and in some cases, impaired ion and neurotransmitter turnover converge in disease. As aforementioned, astrocytic Kir4.1 is also needed for appropriate uptake of perisynaptic glutamate by GLT-1 (Djukic et al., 2007). Expression of GLT-1 is downregulated in post-mortem brains of Huntington’s disease patients (Arzberger et al., 1997). Similarly, expression of Kir4.1 is also significantly reduced in animal models of Huntington’s disease. Excitingly, overexpression of Kir4.1 in striatal astrocytes rescues *Glt-1* expression deficits, improves function of medium spiny neurons (disproportionately lost in Huntington’s disease) and increases animal survival (Tong et al., 2014). As Kir4.1 is affected in a vast array of neurological disorders and modifies multiple critical functions of astrocytes, identifying therapeutic strategies that target Kir4.1 function could have extensive medical impact.

Outside of the CNS, loss of glial support disrupts synaptic maintenance and overall peripheral nerve health. In vertebrates, tSCs are required for maintenance of the NMJ, and loss of tSCs (Santosa et al., 2018) or neuron-glial signaling (Jablonka-Shariff et al., 2020) can result in degeneration. Similarly, post-developmental ablation of *C. elegans* AMsh glia (exhibit ensheathment similar to non-myelinating Schwann cells) results in retraction of neuron receptive endings (pre-synaptic) that sense the environment (Bacaj et al., 2008; Singhvi et al., 2016; Wallace et al., 2016). Disruption of glia-derived ion transporters does not only impact synaptic maintenance but can disrupt peripheral nerve health. Loss of NKCC1 homologs (Na^+^, K^+^, and Cl^–^ cotransporter) from peripheral glia results in abnormal nerve swelling and axonal defasciculation in *Drosophila* (Leiserson et al., 2000, 2011) and zebrafish (Marshall-Phelps et al., 2020), resulting in neuropathy-like symptoms (Leiserson et al., 2000, 2011; Marshall-Phelps et al., 2020). In sum, glia support overall nervous system health by careful management of the extracellular environment.

## Glial Regulation of Nervous System Function

### Metabolic Support of Neurons by Glia

Glia instruct nervous system function beyond the synaptic unit. Neuronal signaling is energetically demanding, yet energy stores (e.g., glucose) within the blood are largely inaccessible to neurons due to blood-brain and blood-nerve barriers (Poduslo et al., 1994; Daneman and Prat, 2015). Both central and peripheral glia, which more directly interact with vasculature, respond to the metabolic states of neurons and provide critical metabolic support for continued nervous system function. This role of glia was first described in the honeybee retina (Tsacopoulos and Poitry, 1982; Tsacopoulos et al., 1987, 1988) and later for Müller glia in the rodent retina (Poitry-Yamate and Tsacopoulos, 1992; Poitry-Yamate et al., 1995). This process converges on a glia-to-neuron lactate shuttle following glycolysis, regardless of system (invertebrate vs. vertebrate) or cell type (Fünfschilling et al., 2012; Lee et al., 2012; Beirowski et al., 2014; Pooya et al., 2014; Volkenhoff et al., 2015; Saab et al., 2016; Philips and Rothstein, 2017; Delgado et al., 2018; Yildirim et al., 2019; Deitmer et al., 2019; Bouçanova and Chrast, 2020). Astrocytes, which form an integral component of the neurovascular unit, are also known to shuttle lactate to neurons in both animal models and in the human brain (Pellerin and Magistretti, 1994; Svichar and Chesler, 2003; Mangia et al., 2009; Deitmer et al., 2019). According to the Astrocyte-to-Neuron Lactate Shuttle hypothesis (Pellerin and Magistretti, 1994), glucose passes through transporters between capillaries and astrocytes; it is ultimately converted from pyruvate to lactate following glycolysis, and transported into neurons through monocarboxylate transporters (MCTs). Once inside the neuron, lactate reverts back to pyruvate via lactate dehydrogenases for use in aerobic respiration within mitochondria (Steinman et al., 2016; Deitmer et al., 2019). This process is elegantly coupled to glutamate release from neighboring synapses, ensuring that energy supply meets demand (Pellerin and Magistretti, 1994). Similarly, blood brain barrier glia in *Drosophila* transfer lactate from the haemolymph to neurons in the CNS via the MCT *chaski*, and loss of *chaski* from glia disrupts excitatory synaptic transmission and locomotion (Delgado et al., 2018).

In CNS white matter and in peripheral nerves, metabolic support of axons is carried out by myelinating oligodendrocytes and Schwann cells, respectively (Fünfschilling et al., 2012; Philips and Rothstein, 2017; Bouçanova and Chrast, 2020). The myelin sheath is organized into multiple functional domains, including cytoplasmic (myelinic) channels that facilitate uptake of metabolites and ions from neighboring astrocytes (for oligodendrocytes) or from the environment (both oligodendrocytes and Schwann cells), which can then be transferred to the associated axonal segment (Lutz et al., 2009; Salzer, 2015; Snaidero et al., 2017). Given that mutations in many genes enriched in myelinating glia (e.g., PLP, CNPase, and MPZ) cause axonal degeneration in the absence of overt dysmyelination (Philips and Rothstein, 2017; Bouçanova and Chrast, 2020), this function of oligodendrocytes and Schwann cells is distinct from the role of myelin in saltatory conduction (discussed below in section “Myelination and Circuit Function”). In the CNS, oligodendrocytes take in extracellular glucose, which is then converted to lactate and transferred to neurons via the monocarboxylate transporter MCT1 (Lee et al., 2012). Accordingly, oligodendrocyte-specific knockout of MCT1 *in vitro* within mouse organotypic spinal cord slice cultures, or *in vivo* within the optic nerve and corpus callosum, causes axonopathy prior to myelin damage (Lee et al., 2012). A recent report found that glucose uptake by oligodendrocytes can be stimulated by binding of glutamate to oligodendrocyte NMDA receptors (Saab et al., 2016). These findings suggest that oligodendrocyte-specific metabolic support, as with astrocytic support, is coupled to neuronal output. In the PNS, disruption of glial metabolism and mitochondrial homeostasis via Schwann cell-specific knockout of the serine/threonine kinase LKB1 causes a severe, progressive axonopathy reminiscent of diabetic neuropathy (Beirowski et al., 2014). Thus, glia ensure energy demand is met for proper nervous system homeostasis.

### Myelination and Circuit Function

Even when ionic and metabolic conditions are met, circuits within the vertebrate nervous system cannot function without myelin. Myelin is a multi-lamellar sheath generated by oligodendrocytes in the CNS and Schwann cells in the PNS (Salzer, 2015; Ackerman and Monk, 2016; Simons and Nave, 2016). Myelin promotes saltatory conduction through insulation of axonal segments and clustering of ion channels at nodes of Ranvier (discussed further in Rasband and Peles, 2015). Myelin thickness is highly stereotyped, and mutations that either increase (hyper) or decrease (hypo) myelin thickness or alter myelin sheath length, can adversely affect action potential propagation (Court et al., 2004; Michailov et al., 2004; Simpson et al., 2013; Li, 2015; Etxeberria et al., 2016; Camargo et al., 2017). These data suggest that myelin state must be tightly regulated. A 3D TEM reconstruction of myelinated segments along pyramidal neurons in the mouse neocortex demonstrated extensive variability in the extent and pattern of myelination along axon tracts, leading to the hypothesis that oligodendrocytes dynamically respond to, and modify, circuit activity (Tomassy et al., 2014). In the past decade, a number of studies demonstrated that neuronal activity tunes the level of oligodendrocyte myelination *in vivo* (Monje, 2018). In zebrafish, silencing of reticulospinal neurons or *phox2b*+ hindbrain neurons via expression of tetanus toxin biased axon selection to electrically active neighbors (Hines et al., 2015; Mensch et al., 2015). Pharmacological activation of reticulospinal neurons also enhanced axon selection, which could be blocked by expression of tetanus toxin. This finding suggests that oligodendrocytes monitor neuronal activity through release of local synaptic vesicles (Mensch et al., 2015). Indeed, a recent report demonstrated that oligodendrocytes express post-synaptic proteins, putative sites of axon-glial communication, which are necessary to tune sheath length and number (Hines et al., 2015; Hughes and Appel, 2019). In mouse, oligodendrocyte myelination is also regulated by neuronal activity (Gibson et al., 2014; Etxeberria et al., 2016; Swire et al., 2019; Bacmeister et al., 2020). Optogenetic activation of deep cortical projection neurons of the premotor cortex is sufficient to stimulate oligodendrocyte precursor cell proliferation, enhance myelination, and improve motor learning (Gibson et al., 2014). In contrast, neuronal silencing by monocular deprivation caused myelin internode length in the optic nerve to be significantly shorter, resulting in reduced nerve conduction velocity (Etxeberria et al., 2016). Together, these data exhibit that myelin is much more dynamic than previously appreciated, and that myelin plasticity can shape circuit function.

### Glia and Neural Circuit Function

Finally, glia instruct circuit function through modulating activity-dependent plasticity mechanisms that function both locally (synapse-level) and globally (circuit-level). As this subject has been reviewed extensively, here we will direct readers to relevant literature (Araque et al., 2014; Wu et al., 2015; Allen and Eroglu, 2017; Salter and Stevens, 2017; Babola et al., 2018; Long et al., 2018; Bar and Barak, 2019; Durkee and Araque, 2019; Foster et al., 2019; Marinelli et al., 2019; Mederos and Perea, 2019; Ackerman et al., 2020), and instead highlight two pertinent examples of astrocyte-mediated neuromodulation in fly (Ma et al., 2016) and zebrafish (Mu et al., 2019). Astrocytes display local and population-wide calcium transients in response to changes in neuronal activity, and it is thought that these transients may in turn influence circuit function (Fatatis and Russell, 1992; Porter and McCarthy, 1996; Nett et al., 2002; Nimmerjahn et al., 2009; Srinivasan et al., 2015). In a landmark study, Ma et al. (2016) identified TRP channel Waterwitch (Wtrw) activity and octopamine-tyramine receptor (Oct-TyrR) signaling as key mediators of astrocyte-dependent neuromodulation. *Drosophila* astrocytes within the ventral nerve cord exhibit oscillatory waves of calcium activity in response to neuronal activity. Here, the authors found that octopamine and tyramine signaling (analogous to vertebrate norepinephrine) from Tdc2+ neurons in the ventral nerve cord activates Oct-TyrR on astrocytes, which in turn allows for calcium entry via Wtrw. Calcium influx drives secretion of an astrocyte-derived cue (potentially ATP) that inhibits adenosine receptors on local dopaminergic neurons, which is necessary for proper larval chemotaxis and startle behavior. Thus, astrocyte-mediated neuromodulation occurs *in vivo*, is necessary for circuit function, and drives animal behavior (Ma et al., 2016).

Until recently, evidence of bona fide astrocytes, with their characteristic stellate structure, was lacking in zebrafish (Lyons and Talbot, 2015; Mu et al., 2019; Chen et al., 2020). Instead, radial glia were believed to perform astrocytic functions in zebrafish (Kroehne et al., 2011; Kyritsis et al., 2012). Excitingly, a recent study showed that a subtype of astrocytes in zebrafish called radial astrocytes are actively engaged in neural computation *in vivo* within a behaviorally relevant circuit. Futility-induced passivity (“giving up”) is a strategy that allows animals to conserve energy following repeated failed attempts to achieve a given task (Maier, 1984; Warden et al., 2012). In this pioneering work, Mu et al. (2019) developed a virtual reality arena that would induce visually evoked, perceived swimming failures. Using whole-brain calcium imaging to simultaneously monitor neuronal and glial calcium levels, they found that repeated swimming failures resulted in accumulation of calcium in radial astrocytes that reside within the lateral medulla oblongata. Localized ablation of radial astrocytes or disruption of astrocytic calcium signaling using an IP3R inhibitor (xestospongin C) strongly suppressed futility-induced passivity, but not wildtype swimming behavior. Through further circuit analysis, the authors demonstrate that radial astrocytes signal via an unknown mechanism to activate downstream GABAergic neurons within the lateral medulla oblongata and suppress continued motor output. Interestingly, accumulation of calcium within radial astrocytes is also dependent on norepinephrine signaling from neurons within the norepinephrine expressing cluster of the medulla oblongata (Mu et al., 2019). Recently, cells resembling mammalian spinal cord astrocytes in every aspect (stellate morphology, marker expression, and calcium signaling) were described in zebrafish for the first time, and were also shown to be norepinephrine sensitive (Chen et al., 2020). These studies together suggest that norepinephrine signaling from neurons may be a common trigger for astrocyte-mediated neuromodulation (Paukert et al., 2014; Ma et al., 2016; Mu et al., 2019).

### Glia and Circuit Dysfunction in Disease

It is evident that glial cells strongly contribute to circuit function. Accordingly, glial cell dysfunction is present in a variety of neurodevelopmental disorders that impact neural network activity (Ma et al., 2016; Mondelli et al., 2017; Salter and Stevens, 2017). This is particularly apparent in neural networks that exhibit higher than average levels of neuronal activity and therefore have greater energetic demands. Motor neurons are highly polarized and energetically demanding cells that extend axons in excess of a meter in length in humans. Accordingly, they require extensive metabolic support from glial cells for continued function and survival (Van Damme et al., 2002; Bélanger et al., 2011; Lee et al., 2012; Morrison et al., 2013; Bouçanova et al., 2020). Amyotrophic lateral sclerosis (ALS) is a progressive neurodegenerative disorder characterized by specific loss of lower and upper motor neurons in the spine and motor cortex, respectively, resulting in paralysis and mortality within ∼5 years of disease onset. Though the majority of ALS cases are non-hereditary (sporadic), 10% of ALS cases are familial in nature and are mapped to known disease-causing loci. Most familial ALS cases are caused by mutations in a handful of genes including SOD1, Tpd43, Fus, and C9orf72 (Robberecht and Philips, 2013). By modeling ALS in animal models using cell-type specific manipulations (Pramatarova et al., 2001; Lino et al., 2002; Beers et al., 2006; Boillée et al., 2006b; Haidet-Phillips et al., 2011; Wang et al., 2011; Kang et al., 2013; Lee and Sun, 2015; Bouçanova et al., 2020), it became clear that glial cells strongly contribute to both disease onset and progression (Boillée et al., 2006a; Philips and Rothstein, 2014; Yamanaka and Komine, 2018; Filipi et al., 2020). For example, in chimeric mice, disease onset is delayed when mutant SOD1 motor neurons are surrounded and supported by wildtype glial cells (Clement et al., 2003).

Analysis of human tissue revealed that microglial and astrocyte populations adopt activated, pro-inflammatory states locally and systemically in ALS (McCombe and Henderson, 2011; Robberecht and Philips, 2013; Blasco et al., 2017; McCauley and Baloh, 2019). This switch in cell state adversely impacts motor neuron survival through the combined cytotoxic effects of inflammation and a loss of homeostatic functions, such as neurotransmitter turnover (Liddelow et al., 2017). Astrocytes are necessary for glutamate clearance from the synaptic cleft (Allen, 2014). Downregulation of the glutamate transporter EAAT2 is evident in animal models expressing mutant SOD1^*G*93A^ (Howland et al., 2002; Boston-Howes et al., 2006), which could be rescued in part via glutamate uptake by focal transplantation of wildtype astrocytes (Lepore et al., 2008). This change in expression causes aberrant accumulation of perisynaptic glutamate, which results in glutamate induced excitotoxicity (Filipi et al., 2020). In the healthy nervous system, astrocytes position mitochondria at places with high energetic demand (Agarwal et al., 2017), because glial transfer of lactate is coupled to extracellular levels of glutamate (Pellerin and Magistretti, 1994). MCT1 expression in oligodendroglia is similarly downregulated in models and patients with mutant SOD1, suggesting that glutamate-induced excitoxicity may be compounded by a lack of glia-mediated metabolic support (Lee et al., 2012). A recent study found that suppressing astrocyte activation and subsequent astrogliosis significantly prolonged the lifespan of a mouse model of ALS (*SOD1^*G*93A^*), implicating gliosis as a putative target for therapeutic intervention (Guttenplan et al., 2020).

## Concluding Remarks on Glia as Therapeutic Targets in Disease

To summarize, the nervous system contains a variety of glial cell types that collectively orchestrate neural development and ensure continued nervous system function. The field of glial biology has long focused on how individual glial subtypes influence neuronal health and longevity. Thus, we have achieved a great deal of success in delineating how disruptions to single glial cell populations contribute to pathology in various animal models of disease. As neurological disease states are often accompanied by dysfunction in multiple glial populations, it is unsurprising that therapeutic interventions that target individual glial and neuronal populations have been met with limited success. Furthermore, it is increasingly evident that in addition to glia-neuron interactions, dynamic glial-glial communication instructs development and function of the healthy and diseased nervous system. Generation of complementary genetic strategies that facilitate independent manipulation of multiple cell populations in vertebrates, while leveraging invertebrate models that are already amenable to complex genetics approaches, is necessary to understand how different glial subtypes function in concert with one another *in vivo.* By expanding the scope of our experiments to account for the complexity of these interactions, we have the potential to uncover novel therapeutic strategies to simultaneously inhibit glial inflammation and promote glial homeostatic functions to drive repair and recovery.

## Author Contributions

SA and VF conceived the topic. IL-B prepared the figures and together with VF and SA and wrote sections “Introduction” to “Glia in Neuronal Migration and Pathfinding of Axons and Dendrites.” SA wrote sections “Glial Regulation of Synapse Numbers” to “Concluding Remarks on Glia as Therapeutic Targets in Disease” with comments from IL-B and VF. All authors contributed to the article and approved the submitted version.

## Conflict of Interest

The authors declare that the research was conducted in the absence of any commercial or financial relationships that could be construed as a potential conflict of interest.
